# Innovation in Gastroenterology—Can We Do Better?

**DOI:** 10.3390/biomimetics7010033

**Published:** 2022-03-19

**Authors:** Eyal Klang, Shelly Soffer, Abraham Tsur, Eyal Shachar, Adi Lahat

**Affiliations:** 1 Department of Diagnostic Imaging, Sheba Medical Center, Tel Hashomer, Israel, and Sackler Medical School, Tel Aviv University, Tel Aviv 6997801, Israel; eyal.klang@mountsinai.org; 2 Sheba Talpiot Medical Leadership Program, Tel Hashomer, Israel, and Sackler Medical School, Tel Aviv University, Tel Aviv 6997801, Israel; 3 DeepVision Lab, Sheba Medical Center, Tel Aviv 6997801, Israel; 4 Department of Population Health Science and Policy, Institute for Healthcare Delivery Science, Mount Sinai Health System, New York, NY 10016, USA; 5 Internal Medicine B, Assuta Medical Center, Ashdod, Israel, and Ben-Gurion University of the Negev, Be’er Sheva 8410501, Israel; 6 Samson Assuta Ashdod University Hospital, Ha-Refu’a St 7, Ashdod 7747629, Israel; 7 Department of Obstetrics and Gynecology, Sheba Medical Center, Tel Hashomer, Israel, and Sackler Medical School, Tel Aviv University, Tel Aviv 6997801, Israel; avi.tsur@sheba.health.gov.il; 8 Department of Gastroenterology, Sheba Medical Center, Tel Hashomer, Israel, and Sackler Medical School, Tel Aviv University, Tel Aviv 6997801, Israel; eyal.shachar@gmail.com (E.S.); zokadi@gmail.com (A.L.)

**Keywords:** innovation, artificial intelligence (AI), virtual reality (VR), telemedicine, microbiome, endoscopy

## Abstract

The health system can reap significant benefits by adopting and implementing innovative measures, as was recently demonstrated and emphasized during the COVID-19 pandemic. Herein, we present our bird’s eye view of gastroenterology’s innovative technologies via utilizing a text-mining technique. We analyzed five research fields that comply with innovation: artificial intelligence (AI), virtual reality (VR), telemedicine, the microbiome, and advanced endoscopy. According to gastroenterology literature, the two most innovative fields were the microbiome and advanced endoscopy. Though artificial intelligence (AI), virtual reality (VR), and telemedicine trailed behind, the number of AI publications in gastroenterology has shown an exponential trend in the last couple of years. While VR and telemedicine are neglected compared to other fields, their implementation could improve physician and patient training, patient access to care, cost reduction, and patient outcomes.

## 1. Introduction

Innovation has multiple definitions in scientific papers and is highly addressed and valued in many life disciplines [1].

Following the Organization for Economic Co-operation and Development (OECD) manual’s definition, innovation is defined as: “the production or adoption, assimilation, and exploitation of a value-added novelty in economic and social spheres; renewal and enlargement of products, services, and markets; development of new methods of production; and the establishment of new management systems. It is both a process and an outcome” [2]. 

Undoubtedly, the health system can benefit immensely from adopting and implementing innovative measures, and the mega-challenges that arose from the Coronavirus disease 2019 (COVID-19) pandemic further emphasized the importance of healthcare innovations. However, in real life, specifically under stressful working loads and sub-optimal physical, economic and regulatory conditions, implementing new working strategies might become exceedingly challenging. 

Illuminating the advances, and on the other hand, the drawbacks in innovation employment may help improve and enhance its use in daily practice. The “text-mining” technique, incited by current computational power and machine learning, enables broad-scale data extraction [3] and can be employed to characterize trends and examine dynamics in a research field [4].

Our aim was to present a bird’s-eye view of innovative technologies’ status in gastroenterology. Our survey focused on five important research fields that comply with innovation: artificial intelligence (AI), virtual reality (VR), telemedicine, the microbiome, and advanced endoscopy.

## 2. Methods

### 2.1. Dataset

The U.S. National Center for Biotechnology Information (NCBI) provides public application programming interfaces (APIs) that allow programmatic access to the PubMed database. We have used the publicly available PyMed Python package to query the PubMed API. 

The following data were extracted for each entry: PubMed unique article ID (PMID), title, publishing journal, abstract text, keywords (if any), and authors’ affiliations. The search was performed on 7 March 2021. Duplicate records (PMID) were omitted.

### 2.2. Inclusion Criteria

The authors (senior gastroenterologists and AI specialists) decided on key terms (Table 1) related to five research fields that comply with innovation: artificial intelligence (AI), virtual reality (VR), telemedicine, the microbiome, and advanced endoscopy. The search was conducted in PubMed entries’ titles and abstracts. Entries were limited to publications between 1 January 2000 to 31 December 2021.

### 2.3. Data Processing

Data processing and result visualization were written in Python (Ver. 3.6.5, 64 bits). Each title, study abstract, and authors’ affiliations were lowercased. 

A list of open-access journals was obtained from the Scimago Journal & Country Rank site (https://www.scimagojr.com/, accessed on 8 March 2022). The list was merged to the PubMed data using International Standard Serial Numbers (ISSN). Gastroenterology and hepatology journals were classified using the merged list.

Annual trends of innovation-related publications between 2000–2021 were plotted for the entire PubMed data set and the Gastroenterology and hepatology sub-group.

## 3. Results 

**Figure 1:**
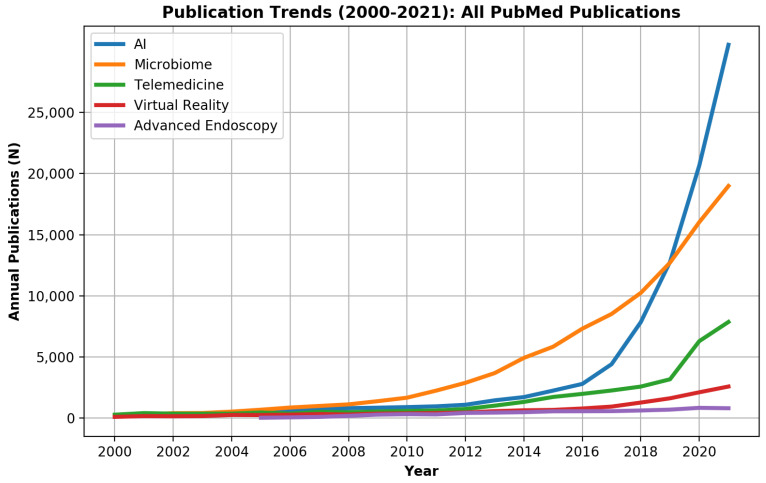
Publication trends of innovation research fields (artificial intelligence (AI), virtual reality (VR), telemedicine, the microbiome, and advanced endoscopy) in the medical field during 2000–2021.

Overall, during 2000–2021, 327,460 PubMed records were innovation-related as defined by the study (Figure 1). Of those, 15,520 (4.7%) records were published in gastroenterology and hepatology journals. 

**Figure 2:**
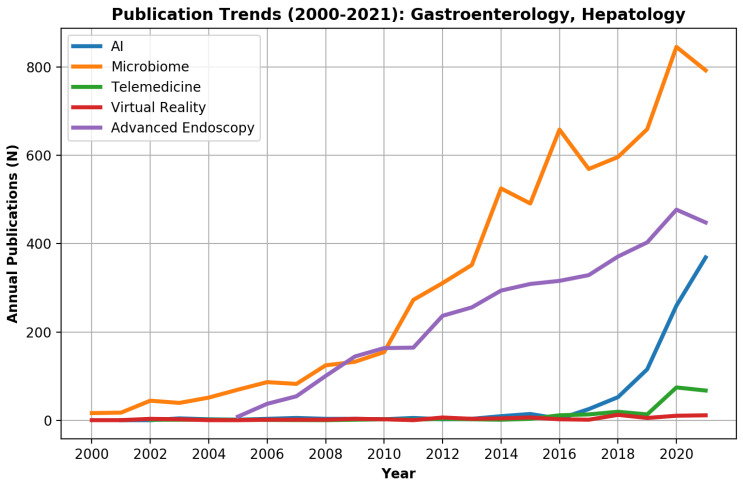
Publication trends of innovation research fields (artificial intelligence (AI), virtual reality (VR), telemedicine, the microbiome, and advanced endoscopy) in the gastroenterology field during 2000–2021.

For the entire PubMed literature, the 2 major innovation fields were the microbiome with 94,993 (29.0%) publications and AI with 82,788 (25.3%) publications (Figure 1). However, AI surpassed microbiome publications during 2019 and showed a marked exponential curve. In gastroenterology and hepatology journals, the microbiome, with 6896 publications (44.4%), and advanced endoscopy, with 4117 publications (26.5%), were the leading innovation fields, while AI showed only a minor rise with 899 (5.8%) publications (Figure 2).

While microbiome- and advanced endoscopy-related publications started to rise in the gastroenterology and hepatology literature during 2000–2008, most digital technology innovation-related publications (AI, VR, telemedicine) were written in the last decade. Telemedicine literature started to rise only in the last two years. 

## 4. Discussion 

The subject of the microbiome shows a high volume of publications in the gastroenterology and hepatology field, which reflects the obvious connection to the GI tract physiology and pathophysiology. Recent research on the microbiome focuses on the effect of specific taxa on the development of various diseases, including inflammatory bowel disease (IBD) and gastrointestinal (GI) malignancies, with an interesting horizon of fecal microbiome transplantation (FMT) as an effective and specific therapy for IBD and a variety of malignant diseases (interestingly, not only GI-related malignancies [5,6,7,8]. 

The gastroenterology-specific advanced endoscopy field is the second most prevalent and correlates with the particular attention and importance attributed to technical endoscopic improvements. Various technical improvements were implemented in recent years, including high-resolution and high-magnification endoscopes that enable improved pathologic detection, high-resolution small diameter probes, high-resolution microendoscopes (HRME), narrow-band imaging etc. [9,10,11,12]. Improvements and research in this field is a common practice, and new technical improvements that enable better endoscopic views and improved endoscope function (including better bowel cleaning) are predicted in the upcoming years [13,14]. 

Focusing on these topics is almost obvious, as these research fields are largely the domain of gastroenterology, and innovations in these fields are predictable.

Similarly to general medicine, the number of AI publications in the gastroenterology literature has increased exponentially in the last couple of years. Currently, we use AI systems for data analysis [15,16,17], for prediction models of disease outcomes, prognosis, and treatment response [18,19,20,21], and for image analysis during live endoscopy [22,23] and post-endoscopy picture analysis [24,25,26]. Intensified collaboration with AI experts can improve the above models using big-data analysis. Thus, we can reach more solid conclusions on a specific case, enhance patient stratification, and provide personalized tailor-made medicine. Improving data analysis using advanced algorithms for free text mining will enable further progress. Furthermore, improved image analysis may offer higher levels of data processing. It might provide accurate pathology analysis in a real-time setting, facilitating decision making and even causing biopsies to become superfluous in selected cases [27]. 

However, telemedicine and VR are still relatively disregarded in our daily practice. The importance of telemedicine during regular patient care was specifically demonstrated during the COVID-19 pandemic, which emphasized the need for remote patient surveillance [28,29].

Telemedicine can widen our viewpoint on various outcome measures usually hidden from medical sight. Thus, telemedicine may indicate impaired daily activities and lifestyle malfunction. Furthermore, it can improve patients’ compliance and enable continuous and enhanced connection with the medical caregivers. This method was specifically useful in chronic patients requiring intensified monitoring [30,31]. Implementing telemedicine might require specific patients, caregiver education and innovations to overcome barriers preventing its frequent use, such as lack of exposure and fear from new technologies [32,33]. With improved data analysis, telemedicine implementation can improve patients’ care and decision making while maintaining regular medical surveillance facing the challenges imposed mainly (but not only) by the COVID-19 pandemic. 

VR has been used for training purposes for decades. Flight simulators are the most known example, and VR is a cornerstone during pilots’ training. VR has been gaining popularity in medical training during the last two decades, as shown in Figure 1. Most relevant data assess surgeon’s training. 

Naturally, VR has major advantages when using a three-dimensional (3D) model for resident training [34,35]. Furthermore, 3D models can assist surgeons in planning and rehearsing future operations and help with patient’s education and understanding of the forthcoming procedure. Other research themes concern patients’ use of VR during rehabilitation due to various medical conditions. Specific interest was attributed to using VR as a distraction technique during pain and chronic pain treatment by inducing neurophysiological changes beyond simple distraction, as in spinal cord injuries and even phantom pains after amputation [36,37,38,39].

In the gastroenterology field, both models are applicable but rarely implemented. VR can be useful during endoscopy training and practicing [40,41], both for training residents and for planning advanced endoscopic procedures. Patients may also benefit from 3D models of upcoming procedures. Regarding patients’ rehabilitation, VR may probably be used for treating GI motility abnormalities, chronic pelvic pain symptoms, etc. Furthermore, implementing VR might reduce pain during endoscopic procedures and spare the need for enhanced sedation.

A new, interesting research question could relate to the issue of whether the adoption of AI and other novelty advancements has occurred more rapidly in other fields of medicine. Our study addressed the gastroenterology field; however, it is likely that other clinical specialties may also face this delay. This issue merits further research.

Our study has a few limitations. This analysis only provides a high-level look at the field. The sheer number of publications prohibits a manual analysis of the records. A list of terms was determined based on current data in the literature and consensus between the authors, who are senior gastroenterologists and AI specialists. However, different terms might have achieved different results. The data were extracted from MEDLINE/PubMed. Other options such as Google Scholar were not included and might have reached different results. The list of journals was extracted from the open-source Journal & Country Rank site database. Other journal databases may have yielded somewhat different results.

## 5. Conclusions

Gastroenterology journals published relatively large amounts of microbiota-themed research before the topic become investigated broadly in biomedical research. Nevertheless, the field of gastroenterology seems to lag almost a decade behind the biomedical sciences as a whole in the adoption of artificial intelligence as the subject or means of research. It appears vital to bridge this gap and also to identify the most novel modalities and topics that may be overlooked or difficult to adopt by gastroenterology researchers today, similar to how AI was overlooked or proved challenging a decade ago. 

## Figures and Tables

**Table 1:** List of terms used to classify entries into five innovation research fields: artificial intelligence (AI), virtual reality (VR), telemedicine, the microbiome, and advanced endoscopy.

Artificial Intelligence	Artificial Intelligence, Machine Learning, Deep Learning, Convolutional Neural Network, Artificial Neural Network, Computer Vision
Telemedicine	telemedicine, telehealth, mobile health, mhealth, virtual care, telemonitoring, telecare
Microbiome	microbiome, microbiota, probiotic, prebiotic
Virtual reality	virtual reality, augmented reality, extended reality
Advanced Endoscopy	peroral endoscopic myotomy, natural orifice transluminal endoscopic surgery, endoscopic submucosal dissection, video capsule endoscopy, robotics in gastrointestinal endoscopy, bariatric endoscopic treatment, confocal laser endomicroscopy
